# Synthesis of molecular imprinting polymers for extraction of gallic acid from urine

**DOI:** 10.1186/s13065-018-0392-7

**Published:** 2018-02-21

**Authors:** Showkat Ahmad Bhawani, Tham Soon Sen, Mohammad Nasir Mohammad Ibrahim

**Affiliations:** 1 Department of Chemistry, Faculty of Resource Science and Technology, UNIMAS, 94300 Kota Samarahan, Kuching, Sarawak Malaysia; 2 School of Chemical Sciences, Universiti Sains Malaysia, 11800 Gelugor, Penang Malaysia

**Keywords:** Gallic acid (GA), Human urine, Molecular imprinting polymers (MIPs), Acrylic acid, Ethylene glycol dimethacrylate

## Abstract

The molecularly imprinted polymers for gallic acid were synthesized by precipitation polymerization. During the process of synthesis a non-covalent approach was used for the interaction of template and monomer. In the polymerization process, gallic acid was used as a template, acrylic acid as a functional monomer, ethylene glycol dimethacrylate as a cross-linker and 2,2′-azobisisobutyronitrile as an initiator and acetonitrile as a solvent. The synthesized imprinted and non-imprinted polymer particles were characterized by using Fourier-transform infrared spectroscopy and scanning electron microscopy. The rebinding efficiency of synthesized polymer particles was evaluated by batch binding assay. The highly selective imprinted polymer for gallic acid was MIPI1 with a composition (molar ratio) of 1:4:20, template: monomer: cross-linker, respectively. The MIPI1 showed highest binding efficiency (79.50%) as compared to other imprinted and non-imprinted polymers. The highly selective imprinted polymers have successfully extracted about 80% of gallic acid from spiked urine sample.

## Introduction

Gallic acid (GA) is a polyphenolic naturally occurring compound in fruits such as blueberries, strawberries, apples, and bananas or other variety of plants and herbs such as oak bark, tea leaves and witch hazel. Gallic acid is diversely used in various applications because of various pharmacological properties like antitumor and anti-inflammatory [1]. Gallic acid is main member of the polyphenolic family that provides vital antioxidant properties [2]. The extensive usage of gallic acid made an emphases on the researchers to design and develop new materials and/or approach for monitoring GA from different real samples. Molecular imprinting technology is a promising approach for the monitoring of gallic acid in real samples.

Molecularly imprinted polymers are the cross-linked polymeric materials and are able to resist chemical and physical stresses such as organic solvents, heat, acid, bases and others [3]. The concept of polymer that can selectively recognize desired molecules have captured many attentions from scientific community over recent years. These recognition systems in polymers are analogue of the biological recognition systems in the body such as enzymes, DNA, antibodies and aptamers. The imprinted polymers produced from the polymerization process have cavities that can complement to the shape of the desired molecules. The developments in molecular imprinting polymers as chromatography stationary phases especially in high performance liquid chromatography have been driven by the advantage of physicochemical stability and high selectivity in the polymers [4].

The three binding approaches have been used in the synthesis of MIPs such as, covalent method, non-covalent method and semi-covalent method. The most widely used is the non-covalent approach. In non-covalent imprinting method, templates bond to monomers with a non-covalent intermolecular bonding which can be destroyed and created easily. Weak metal coordination, electrostatic interactions, hydrogen bonds and hydrophobic interactions are included in non-covalent forces used by both molecules of chemically and geometrically complement to each other [5]. Simple diffusion can be used to remove templates once polymerized with a polar or acidic solvent and is enough to destroy the non-covalent interaction between template and polymer [6]. While covalent imprinting is less economical and usually troublesome, in this research, all polymers were synthesized by non-covalent approach by precipitation polymerization method.

## Materials and methods

### Materials and reagents

Gallic acid (GA) and 2,2′-azobis(isobutyronitrile) (AIBN) were obtained from R & M Marketing company located in Essex, United Kingdom, acetonitrile was obtained from Avantor Performance Materials Incorporated located in Phillipsburg, New Jersey, acrylic acid (AA), ethylene glycol dimethacrylate (EGDMA) and syringic acid were obtained from Sigma-aldrich Corporated located in St. Louis, Missouri, acetone was obtained from HmbG Chemicals company located in Hamburg, Germany and methanol and acetic acid was obtained from R & M Marketing company located in Essex, United Kingdom.

### Instruments/equipment’s

Fourier-transform infrared spectroscopy (FTIR) (Nicholet iS10), scanning electron microscopy, (SEM) (JEOL JSM 6930 LA), sonic bath (Branson 2510), shaker (Multi Shaker NB-101MT), high-performance liquid chromatography (HPLC) (Model Shimadzu LC-20), and water bath (Model Memmert W350T).

### Preparation of imprinted and non-imprinted polymer beads

The polymers were synthesized by using precipitation method of polymerization. Initially, 1 mM template of gallic acid (GA) was dissolved in 75 mL of acetonitrile followed by the addition of 4 mmol of functional monomer acrylic acid (AA), 20 mM of cross-linker ethylene glycol dimethacrylate (EGDMA) and 30 mg of an initiator 2,2′ azobis(isobutyronitrile) (AIBN) in the same reaction flask. The pre-polymerization solution was sonicated for 10 min followed by the purging of nitrogen gas for 15 min in an ice bath. The reaction flask was then sealed tightly and kept in a water bath for polymerization. The polymerization reaction was carried out initially at 70 °C for 2 h and then temperature was increased and kept constant at 80 °C for next 4 h. The synthesized beads were centrifuged and collected after washing with methanol. The composition of other imprinted polymers is given in Table 1 and were prepared following the same procedure. The non-imprinted polymer was prepared without template molecule.

**Table 1:** Composition of polymers for synthesis

Experiment	Ratio			
	Template, GA (mM)	Monomer, AA (mM)	Cross-linker, EGDMA (mM)	Initiator, AIBN (mg)
MIPI1	1	4	20	30
MIPI2	1	6	28	30
MIPI3	1	12	28	30
MIPI4	1	6	20	30
NIPN1	–	4	20	30

### Extraction of template from the polymer matrix

Extraction of template from the imprinted polymer beads was carried out by washing with mixture of methanol and acetic acid (9:1, v/v). The extraction of template was monitored by using HPLC. This process was repeated until template was not detected by HPLC.

### Characterization of polymer beads

Morphology of polymers surface was observed with SEM coated with gold under reduced pressure. Polymer samples were dried in a vacuum oven at 60 °C for 6 h until constant weight is achieved before analysis. Then, polymer samples were analysed at 32 scans by FTIR.

### Binding capacity

Polymer particles were dried in a vacuum oven at 60 °C for 6 h until constant weight was achieved before adsorption or desorption measurements. A series of conical flasks were used and labelled as MIP1, MIP2, MIP3, MIP4 and NIP1. A 50 mL of feed solution (0.2 mM GA in acetonitrile) at room temperature (26 °C) was taken in five different flasks followed by addition of 500 mg of polymer beads (MIP1, MIP2, MIP3, MIP4 and NIP1). After that conical flasks were agitated on a shaker for 180 min. The samples were collected at different time intervals (0, 30, 60, 90, 120, 150 and 180 min). The collected samples were then analysed by HPLC. High performance liquid chromatography (HPLC) was performed by using acetonitrile, water, acetic acid (60:39.5:0.5) as an eluent and C18 column as a stationary phase. The flow rate of sample was 1.0 mL/min and wavelength for analysis was 268 nm. The extraction percentage (%) of imprinted polymer beads and non-imprinted polymer beads was calculated by the following Eq. 1.

1 $$\text{Extraction}\;\text{percentage} = \frac{{ ( {\text{C}}_{i} - {\text{C}}_{f} )\times 1 0 0 {\text{\% }}}}{{{\text{C}}_{i} }}$$

where C_*i*_ and C_*f*_ are the initial and final concentration of GA in the feed solution, respectively.

### Competitive binding capacity

The competitive binding test was performed by using gallic acid along with syringic acid. In this study, 500 mg of both MIPI1 and NIPN1 polymer beads were immersed in two different flasks containing 30 mL of feed solution (10 ppm of both GA and syringic acid). The reaction flasks were agitated on shaker followed by the same procedure of batch binding. The collected samples were analysed by HPLC. The extraction percentage (%) of MIPI1 polymer beads and NIPN1 polymer beads was calculated by using Eq. 1.

The distribution coefficient, K_*d*_ (mL/g) was calculated by using Eq. 2 as follows:

2 $$K_{d} = \frac{{ ( {\text{C}}_{i} - {\text{C}}_{f} )\times {\text{V}}_{s} }}{{ {\text{C}}_{f } \times {\text{m}}_{p} }}$$

where C_*i*_ (g/L) and C_*f*_ (g/L) are the initial and final concentration of same component, m_*p*_ (g) is the amount of polymer beads, and V_*s*_ (L) is the volume of the feed solution [7].

The selectivity coefficient (k_*GA*-*C*_) was calculated by using Eq. 3 as follows:

3 $$\text{k}_{GA - C} = \frac{{{\text{K}}_{d,GA} }}{{{\text{K}}_{d ,C} }}$$

where K_*d,GA*_ and K_*d,C*_ are the distribution coefficient of gallic acid and syringic acid, respectively [7].

According to Nicolescu et al. [7], relative selectivity coefficients, K′ can be calculated by using Eq. 4.

4 $${K^{\prime} = }\,\frac{{{\text{k}}_{MIP} }}{{{\text{k}}_{NIP} }}$$

where k_*MIP*_ and k_*NIP*_ are the distribution coefficient of imprinted polymers and non-imprinted polymers, respectively.

### Spiking of human urine and extraction efficiency

Firstly, urine was collected from a drug free human. Prior to spiking, urine was filtered and kept in a refrigerator. The spiked human urine was prepared by adding a 30 mL of 10 ppm of GA solution to a 30 mL of human urine. After that 500 mg of MIP I1 and NIPN1 were added into the two different flasks containing spiked human urine. The extraction efficiency was obtained by following the batch binding process and calculated by using Eq. 1. The analysis was performed by using HPLC.

## Results and discussion

### Synthesis of imprinted polymer beads and non-imprinted polymer beads

The polymeric microspheres with adequate control of product morphology can be achieved by using precipitation polymerisation [8]. It produces microspheres with smooth and clean surfaces and gives suitable particle sizes. This method is simple and have many advantages because stabilisers are abandoned during the polymerisation process as compared to suspension polymerisation. On the other hand, non-covalent approach has been adopted during polymerization process. In this study five different polymers varying in chemical composition have been synthesized by precipitation polymerization using non-covalent approach.

### Morphology of polymer beads

**Fig. 1:**
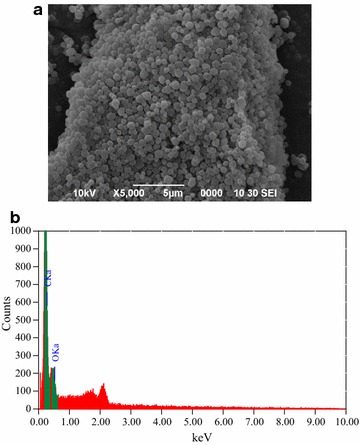
**a** SEM of MIP. **b** EDS analysis of MIP

The scanning electron microscope (SEM) was used to study the morphology of synthesized beads. The SEM micrograph (Fig. 1a) revealed that the microsphere beads were produced during the polymerization process. The obtained polymeric beads are spherical in shape and with an average size of 0.85 µm. The morphology of polymer beads is greatly influenced by the method of polymerization and the solvents used in the synthesis.

A quantitative energy dispersive X-ray (EDS) analysis was performed to establish amount of main chemical elements such as carbon (C) and oxygen (O) present in the imprinted polymer. It was (Fig. 1b) found that the significant amount of carbon (67.92%) and oxygen (32.08%) were present in the sample.

### FTIR characterisation

**Fig. 2:**
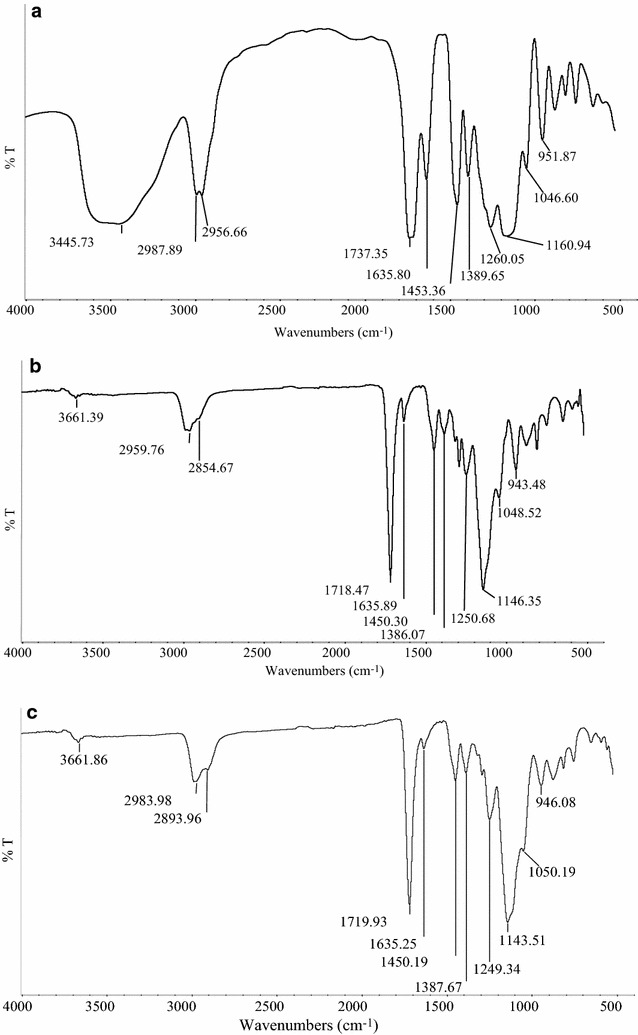
**a** FTIR of MIPI1. **b** FTIR of MIPI2. **c** FTIR of MIPI3

**Fig. 3:**
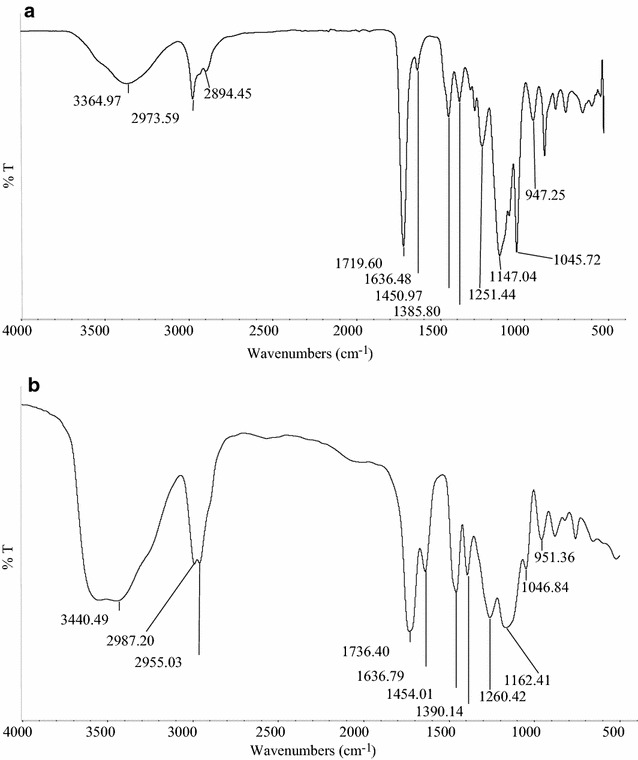
**a** FTIR of MIPI4. **b** FTIR of NIPN1

The Chemical structure of MIPs and NIP (Figs. 2a–c, 3a, b) was examined by using the fourier transform infrared spectroscopy (FTIR). The FTIR analysis of all the polymers is summarized as follows. A strong broad peak at ~ 3600–3200/cm is attributed to the stretching vibration of O–H bond. There are also strong peaks observed at 2987.89–2854.87/cm due to the C–H stretching. A strong peak at ~ 1737–1718 cm is attributed to the vibration mode of C = O which was observed in the IR spectra of the MIPs as well as NIP. Besides that, there is a sharp band at ~ 1635.80 cm which indicated the presence of C=C of alkene in the spectrum. The two peaks located in the range of 1453.36–1385.50/cm are due to the presence of –C–H bending. The peaks observed between 1260.05 and 1046.60/cm indicated the presence of O-C stretching vibration. The peak at 951.87/cm is due to the stretching of =C–H and =CH_2_.

### Binding capacity

**Fig. 4:**
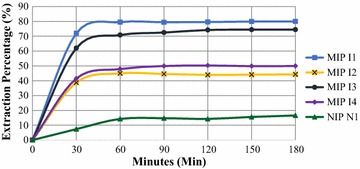
Binding capacity of polymer beads

The re-binding efficiency of imprinted polymers was estimated by using batch binding study. It was observed that MIPI1 has highest rebinding efficiency as compared to other MIP’s. From the Fig. 4 it is clear that the highest rebinding efficiency was achieved at 60 min time and after that it remained almost constant. The efficiency in case of NIP was lowest, this may be because of the absence of complementary sites. Yan and Row in 2006 [9] reported that the imprinted polymer have permanent cavity for the template and therefore imprinted polymer will selectively bind with template molecule. According to Bergmann and Peppas 2008 [6], these cavities not only sustain the shape of the desired template but also sustain the chemical functionalities from the complementary template. The low efficiency of MIP’s (MIPI2, MIPI3 and MIPI4) may be due to the presence of scattered cavities in the polymer matrix [10]. The scattered binding sites have low rebinding affinity for the template molecule.

### Competitive binding capacity

The Specific GA binding by imprinted beads as compared to non-imprinted beads can be expressed by competitive adsorption test [7]. The competitive binding of GA was evaluated with syringic acid as a competitive template. Syringic acid was selected as competitive template because it belongs to the same group of phenolic acids with GA. In this test, two compounds (GA and syringic acid) were tested using both MIPI1 and NIPN1. The selective binding of GA and syringic acid was evaluated by using RP–HPLC measurements. The distribution ratio of GA in both MIPI1 and NIPN1 was higher as compared the distribution ratio of syringic acid in both MIPI1and NIPN1. This results in higher selectivity coefficient of GA as compared to syringic acid in both MIPI1 and NIPN1 (Table 2). The results indicated that the imprinted polymer has got complimentary binding sites or cavities with the GA as compared to syringic acid.

**Table 2:** Specific parameters of polymers for the competitive uptake from feed solution

Polymer code	K_*d*,*GA*_ (mL/g)	K_*d*,*C*_ (mL/g)	k_*GA*-*C*_	k′
MIPI1	8.67	3.63	2.39	2.07
NIPN1	5.28	4.57	1.16	–

## Application

The extensive use of GA for many pharmacological purposes is the main reason to synthesize selective imprinted polymers for the extraction of GA from urine. The MIPI1 was used for the extraction of GA from urine and it was found that about 80% of GA was successfully extracted from the spiked urine sample.

## Conclusion

The imprinted polymers for GA were prepared by precipitation polymerisation method using non-covalent approach. The selected MIPI1 have successfully extracted 80% of GA from the spiked urine sample. This could open diverse applications of imprinted polymers for the extraction of compounds from various biological samples.
